# Effects of Different Undersizing Site Preparations on Implant Stability

**DOI:** 10.3390/ijerph17238965

**Published:** 2020-12-02

**Authors:** Bernardo Ferreira Lemos, Paula Lopez-Jarana, Carlos Falcao, Blanca Ríos-Carrasco, Javier Gil, José Vicente Ríos-Santos, Mariano Herrero-Climent

**Affiliations:** 1 Faculty of Health Sciences, Fernando Pessoa University, 4249-004 Porto, Portugal; blemos@ufp.edu.pt (B.F.L.); cfalcao@ufp.edu.pt (C.F.); 2 Porto Dental Institute, 4150-518 Porto, Portugal; plopezjarana@hotmail.com (P.L.-J.); dr.herrero@herrerocliment.com (M.H.-C.); 3 Department of Periodontology, University of Seville, 41009 Seville, Spain; brios@us.es; 4 Faculty of Dentistry, Bioengineering Institute of Technology, International University of Catalonia, 08017 Barcelona, Spain; xavier.gil@uic.es

**Keywords:** dental implant, underpreparation, implant preparation, implant stability, insertion torque, ISQ, RFA

## Abstract

As immediate loading protocols are becoming more frequent, the primary stability of implants has become an essential criterion for the osseointegration of dental implants. Based on this, the objective of this study was to understand the influence of different undersized surgical preparation sites on the insertion torque (IT) and implant stability quotient (ISQ). Four different site-preparation protocols were performed on fresh humid type III bovine bone: one control, the standard protocol recommended by the manufacturer (P1), and three variations of undersized techniques (P2, P3 and P4). The implant used was VEGA by Klockner Implant System. The sample size was n = 40 for each of the four groups. A torquemeter was used to measure the IT, and the ISQ was measured with a Penguin RFA. Both variables showed a tendency to increase as the preparation technique was reduced, although not all the values were statistically significant (*p* < 0.05) when comparing with the standard preparation. The preparations without a cortical drill, P2 and P4, showed better results than those with a cortical drill. Given the limitations of this study, it can be concluded that reducing the implant preparation can increase both the IT and ISQ. Removing the cortical drill is an effective method for increasing implant stability, although it should be used carefully.

## 1. Introduction

The primary stability of dental implants is a key element in obtaining successful osseointegration [1]. It is described as the frictional force of contact between the implant surface and the drill bone preparation after its insertion [2]; it is a mechanical concept. Clinically, it is defined as the non-existence of mobility, where the implant stays in the desired final position. The absence of mobility is an important mechanical prerequisite, because implant micromovements greater than 150 µm [3] may generate fibrointegration and induce implant failure [4]. Recent research showed that primary stability could be affected by several factors: the macro-implant design, bone quality, implant size, osseous morphology of the surgical site, drilling protocol technique [5] or surgeon’s skills.

Primary stability was initially evaluated using invasive methods, such as assessing the insertion and disinsertion torque of the implant with a torque gauge or torque wrench during implant insertion [6]. Another noninvasive method, resonance frequency analysis (RFA), was also employed because of its reliably, repeatability and relation with micromovements [7,8,9,10]. However, both techniques were confirmed to analyze different mechanical aspects of the implant stability concept [11]. The first method, assessing the insertion torque (IT), evaluates the resistance to a rotational force against the implant when it is inserted into the drill bone preparation [12]. Each implant manufacturer has their own recommendations about the maximum insertion torque values that are supported by implants.

The RFA system was developed by Meredith in 1996 [13]. This methodology is based on a frequency signal transmitted to a screwed transducer, which produces the vibration of the implant. The RFA is expressed on the centesimal Implant Stability Quotient scale (ISQ), a frequency scale in kHz units ranging from 1 to 100. The greater the stability, the greater the ISQ value [14]. The RFA system is used to measure implant stability as a function of the stiffness of the bone-implant relationship [15]. There is no stability evaluation system that has been demonstrated as the gold standard, with both insertion-torque assessment and RFA being widely used in clinical practice and in many studies [16]. Therefore, the primary stability allows the osseointegration of the implant and bone formation around its surface, strengthening the bone-to-implant contact to provide the secondary stability or biological stability of the implant [17].

Primary stability is considered a prerequisite for the long-term success of immediate or early loading implants [18], and certain clinical factors can limit it [19]. The mechanical force resistance of bone, directly related to its quality, is a determining prognostic factor for the success of implant survival, and one of the essential factors for achieving osseointegration [20]. Poor bone quality, such as type III or IV (of Lekholm and Zarb in 1985) [3], describes a bone volume mainly made up of cancellous bone, with a thin cortical layer. This low-density bone could reduce the success of implant osseointegration in immediate implants or immediate loading protocols [21].

Some authors refer to the clinician’s ability to adjust the surgical technique according to their experience, during the surgical procedure of implant placement, as an important factor for implant stability [22,23].

Several modifications of surgical techniques have been proposed to improve the success of implants located in areas of low bone density [24], aiming to increase the stability after insertion. The proposed techniques are under-drilling or preparation with drills with a diameter smaller than the diameter of the implant, bone condensation or bi-cortical fixation [25].

There are some factors that determine the ideal surgical technique: osteotomy drill preparation, adequate initial implant stability, and avoiding excessive heating or compressive trauma [6]. Primary stability is achieved through mechanical interlocking between the implant and the bone wall of the osteotomy [4].

The proper final osteotomy dimension should be determined based on the bone characteristics via so-called adapted drilling; that is, when selecting the final osteotomy dimension, a different degree of bone compression and primary stability at implant placement can be obtained [26].

To achieve higher values of primary stability, some studies recommend under-drilling or lower dimensions. Depending on the bone characteristics at the planned implant position, an adaptive drilling procedure can be chosen to optimize implant stability [24].

Some studies recommend undersized drilling techniques with the use of a final drill with a smaller diameter (than that recommended in the drilling sequence by the implant manufacturer) than the one corresponding to the implant diameter, to locally optimize bone density, which subsequently improves primary stability at sites with poor bone quality [27].

Others authors propose bone condensation by the use of “condensers” after the pilot drill, to displace the bone into the periphery [5], while others have studied the effect of implant bed preparation without profiling and tapping, with controversial results regarding the resulting bone implant contact [23,28].

The aim of this study was to compare different site preparation techniques and evaluate the influence on implant stability according to the insertion torque and ISQ values from resonance frequency analysis.

## 2. Materials and Methods

In this study, fresh bovine kneecaps wide in diameter were used, which are classified as type III in terms of bone density according to the classification of Lekholm and Zarb, owing to their minor portion of cortical bone and greater medullar proportion [29].

The implant used was the VEGA implant from the Klockner Implant System (SOADCO S.L., Andorra). It is a bone-level-type implant, for use with the switching platform concept, and therefore a two-piece implant with a transepithelial abutment that is connected to the implant by a morse cone-type connection.

The implants used were 3.5 and 4.0 mm in diameter and both 10 mm in length. The surface used was shot blasted with alumina particles and acid passivate, with a roughness (Ra) of 1.30 ± 0.23 µm.

N Query Advisor v4.0 was used to calculate the number of samples based on the study of Herrero-Climent (2012 and 2013). The calculated sample size was n = 18 for *p* < 0.05, but the authors decided to increase it to n = 40, looking for statistical significance. An ethics committee was not necessary, as it was an in vitro study with fresh bovine bone [7,30].

All the procedures were performed in a room at 22 °C. The bone fragments to be used were kept for one hour at room temperature before the procedure once removed from the refrigerator where they were stored.

The implant sites were placed by a single investigator, a surgeon with more than five years’ experience and a broad knowledge of the Klockner Implant System. With the goal of simulating the usual clinical situation, the preparation was performed with an electronic surgical unit (W&H) Dentalwerk, Austria) with physiological serum stored at a temperature of 6 °C.

### 2.1. Implant Stability Measurements

Two variables were measured in order to evaluate implant stability: the resonance frequency and insertion torque (IT). Once the bone sites were prepared for the implants, the latter were inserted mechanically, using the surgical unit, up to half their length. A torquemeter, Tohnichi—ATG6C (Tohnichi Mfg. Co Ltd., Tokyo, Japan), was used to insert the rest of the body at the implant site, and then, the final insertion torque was measured.

The measurements of resonance frequency were performed with the Penguin RFA (Integration Diagnostics, Gothenburg, Sweden). For the RFA values, recorded as ISQ (implant stability quotient) values, two perpendicular measurements were registered. For RFA registration with the Penguin RFA system, a transducer element (MultiPeg) had to be connected to the implant, this being specific for each of the implants to be registered. The two RFA measurements to be performed on each implant were perpendicular to each other. They were performed according to the manufacturer’s suggested technique, bringing the probe perpendicular to the MultiPeg until the warning sound for the registration was perceived. Subsequently, the average value obtained for the two measurements of each implant was considered as the ISQ value. For the VEGA 3.5 mm diameter implant, the MultiPeg used was number 57, ref. 55065, and for the VEGA 4.0 mm, the number was 26, ref. 55034.

### 2.2. Implant Site Preparation

**Figure 1:**
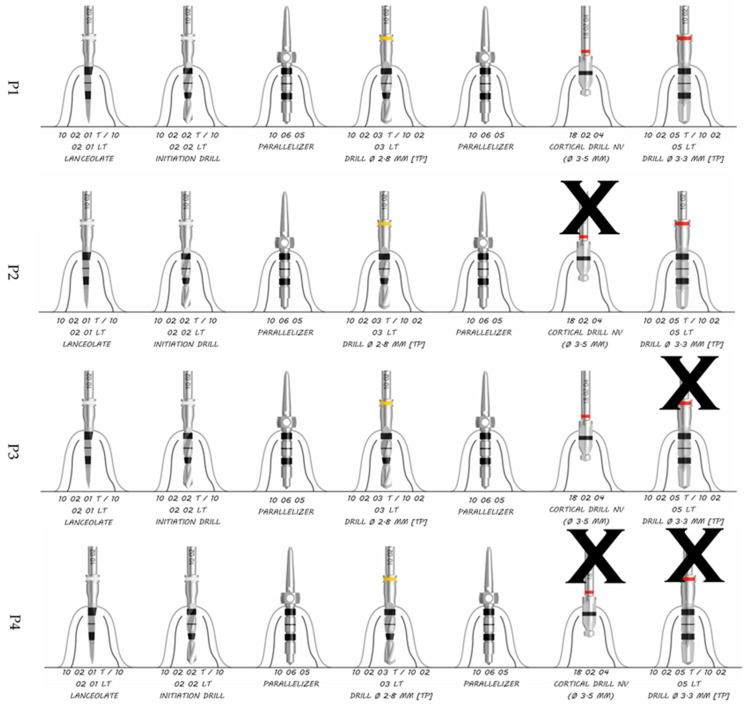
P1, P2, P3 and P4 site preparation sequence for 3.5 mm diameter.

Four different types of implant site preparations were performed (Figure 1): one was the control group (P1), and the others were different variations of implant site underpreparation (P2, P3 and P4). Therefore, the implant surface in contact with the bone differed depending on the type of preparation.

### 2.3. Study Groups

- **P1**—Recommend by the company—control group.
- **P2**—The same as P1 excluding the cortical drill (ref nº 10 02 04).
- **P3**—Horizontal undersized preparation technique that did not include the last full-length drill (ref nº 10 02 05 T/10 02 05 LT) but included the cortical one.
- **P4**—Horizontal undersized preparation technique like P3 but excluding the cortical drill (ref nº 18 02 04).

The implant sites were prepared in such a way that they should have been 1.0 mm subcrestal once inserted.

The preparation sequence proposed by the manufacturer is (Figure 1, first sequence):

- Lanceolate drill for the first 6 mm of preparation or decortication (ref nº 10 02 01).
- Initiation drill with 2.35 mm diameter (ref nº 10 02 02).
- Initiation drill with 2.80 mm diameter (ref nº 10 02 03).
- Cortical drill with 3.5 mm diameter (ref nº 18 02 04).
- Pilot drill with 3.5 mm diameter (ref nº 10 02 05).

**Figure 2:**
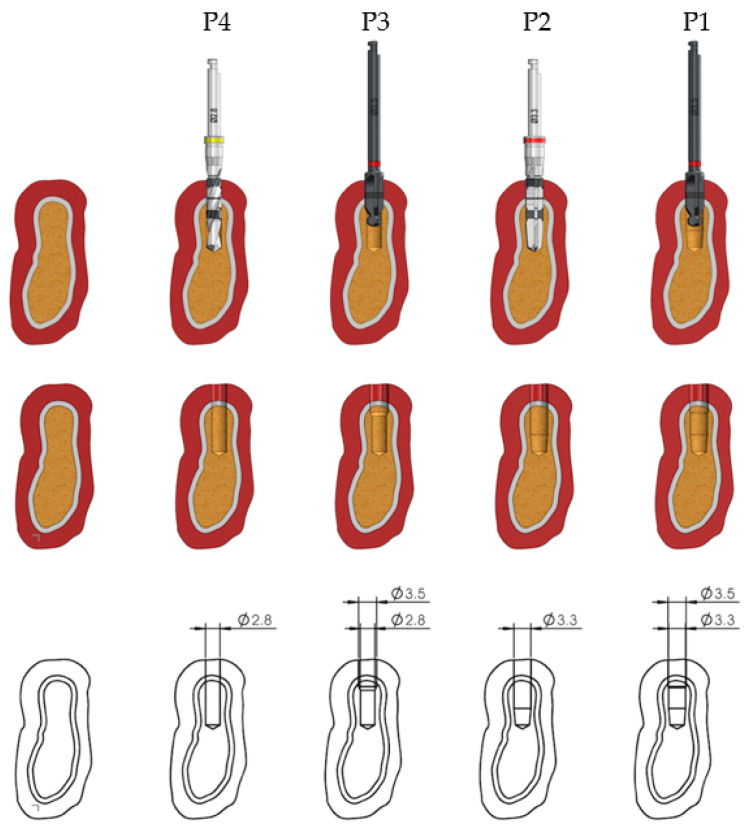
Surface morphology of area of the implant in contact with the bone, and the diameter in each site preparation for 3.5 mm diameter implants.

**Figure 3:**
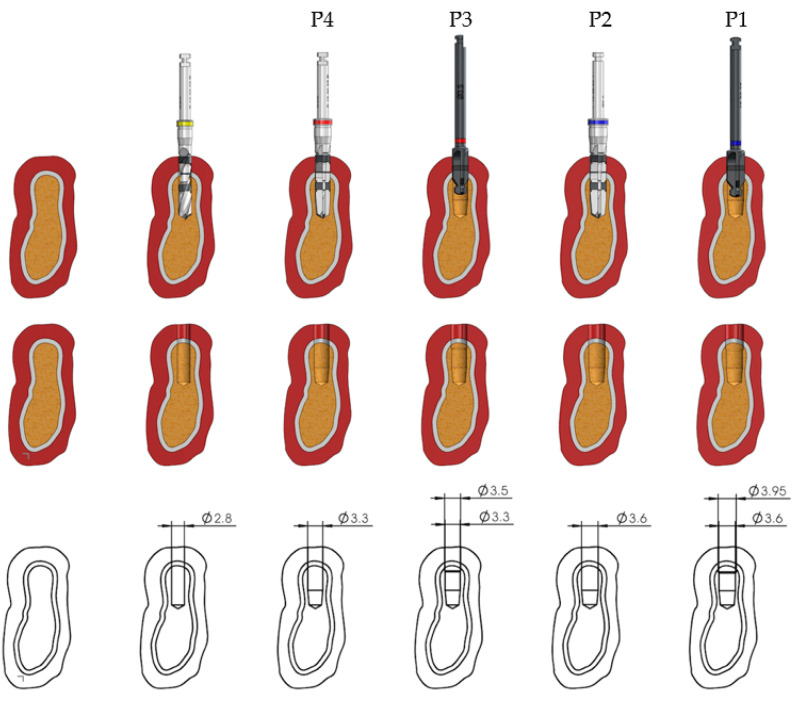
Surface morphology of area of the implant in contact with the bone, and the diameter in each site preparation for 4.0 mm diameter implants.

The drilling sequences to be evaluated in the present study are shown in Figure 1 and Figure 2 for the 3.5 mm diameter implants. Preparation for 4.0 mm diameter implants requires the use of a 3.6 mm diameter pilot drill and a 3.95 mm cortical drill after the use of the 3.3 mm diameter pilot drill; the rest of the sequences are the same as those for implants 3.5 mm in diameter (Figure 3).

Each type of implant bed, depending on each type of preparation, results in a different implant surface in contact with the bone tissue. As observable in Table 1, when there is an undersizing of the implant preparation, the total bone surface of the implant site is decreased, and at the same time, there is an increase in the implant surface in contact with the bone.

### 2.4. Statistical Analysis

The Minitab 16 statistical software was used to determine if there were statistically significant differences for the different variables studied.

When the values exhibited a normal distribution (Shapiro–Wilk test), the statistical analysis was performed using Student’s parametric *t*-test.

When the values did not show a normal distribution, it was performed using the non-parametric Mann–Whitney test.

## 3. Results

During the implant insertion procedure, all the implants reached their final positions without any particular problems.

Table 2 shows the mean values of the IT and ISQ, standard deviations and *p* values, for both the 3.5 and 4.0 mm implants recorded with the four different site-preparation techniques.

As can be seen in Table 2, for the 3.5 mm diameter implants, the IT values in all cases in which any sequence with underpreparation was performed (P2, P3 and P4) were higher than those for cases in which the standard sequence proposed by the manufacturer was performed (P1).

For implants with a diameter of 4 mm, the IT values were higher in those sequences in which underpreparation was performed without the use of the cortical drill (P2 and P4).

When evaluating the mean RFA (ISQ) values, for both the 3.5 and 4.0 mm diameter VEGA implants, the stability values were higher when underpreparation was performed than when the manufacturer’s proposed sequence was used.

When the RFA measurements were analyzed independently, for the first (RFA ISQ A) and second RFA measurements (RFA ISQ B), higher ISQ values are observed for the implants with diameters of 3.5 mm in the underpreparation sequences that did not use the cortical drill. We found a similar situation when analyzing the 4 mm diameter implants, although the differences between the different preparation sequences were smaller.

## 4. Discussion

The increasing demands, from both patients and clinicians, for more predictable and immediate treatments has led to an evolution in treatments making it possible to achieve better primary stability. One of the easiest and most efficient ways of improving primary stability is the underpreparation of the implant site [31,32]. Although it seems to be a predictable method, it should be taken into account that there is an increased potential for the overcompression of the crestal bone, resulting in necrosis. To prevent this from happening, there should be a correct evaluation by the clinician of the bone density [33,34].

In most of the existing literature, the undersizing has been measured by the percentage of reduction of the pilot drill, so most studies do not include underpreparation by the use of the cortical drill like the present study. In the present research, the percentage of the implant surface in contact with the bone tissue once the implant site was ready was calculated, so when comparing the preparation technique recommend by the manufacturer, P1, to the other three undersized ones, P2, P3 and P4, the implant surface had a 23–145% increase in bone contact. The results show that when the implant site was undersized, there was an increase in the RFA values, so micromovements of the implant were decreased and higher primary implant stability was obtained.

Those RFA values were statistically significantly higher in almost every comparison between two implant site preparations (*p* value < 0.05) (Table 3). The ISQ and IT values showed a tendency to increase as there was a reduction of the preparation technique, although there were two values that did not correspond with this tendency, which could be because of the variety of densities in the same piece of bovine bone. These values were found in the 3.5 mm diameter P2, with an IT value of 27.7 ± 16.7, and in the 4.0 mm diameter P3, with an IT value of 34.6 ± 16.3. Those data are consistent with a previous in vitro study by Degidi et al., 2015, which evaluated two different levels of underpreparation (10% and 25%) on fresh humid bovine bone, but instead of varying the set of burs, the authors varied the implant diameter; the results showed that there was an increase in ISQ with both underpreparations when comparing to the standard one (ISQ standard: 69.35 ± 7.35; ISQ with 10% reduction: 73.4 ± 2.33; ISQ with 25% reduction: 72.3 ± 6.3), although only the 10% reduction underpreparation produced a statistically significantly higher ISQ when compared to the standard one [24].

Alghamdi et al., 2011, in a clinical study, compared implant stability by measuring the IT and ISQ for two types of drilling techniques. They placed 52 implants on 29 patients, always controlling the bone density according to radiographic assessments and clinical evaluations, and they ensured that the implants were always placed on low-density bone (types III and IV, according to the Lekholm and Zarb classification). The conventional drilling technique was finished with a 3.5 mm pilot drill for a 4.1 mm diameter implant, and the undersizing drilling technique was finished with a 2.8 mm pilot drill for the same implant diameter. The undersizing drilling technique produced higher values of both IT (35.19 ± 4.79) and ISQ (68.58 ± 4.81) when compared with the conventional drilling technique’s IT (34.62 ± 5.82) and ISQ (66.69 ± 5.41). Although these values were not statistically significant, a tendency of increasing implant stability on low-density bone with this undersizing drilling technique was observed [27]. Results similar to those were found in the present work.

Tabassum et al., 2010, showed higher IT values in situations where underpreparations were performed to prepare the implant site. The study was carried out in epoxy resin models that simulated bone densities of 0.48 g/cm^3^, with different thicknesses of cortical bone. The data were in line with those obtained in the present study, although with differences in the model used for the assessment [35].

Ahn et al., 2012, in an in vitro study, in which solid rigid polyurethane blocks were used as an alternative to bovine low-density bone, they placed 30 implants using three different preparation techniques implants and measured the IT, RFA (ISQ values) and removal torque (RT). The techniques were a standard drilling technique, undersizing preparation and osteotome preparation. They found that the undersized protocol yielded a statistically significantly higher mean IT of 104.57 ± 18.6 Ncm, when compared to the standard protocol, which yielded a mean IT of 89.45 ± 10.03 Ncm. Although no increase in ISQ values was found with undersizing, these IT results matched the tendency found in this research: as the undersizing of the implant site increased, there was a tendency towards increased IT values [5].

Bilhan et al., 2010, showed results similar to those found in this study, showing statistically significantly higher IT values with underpreparation, while the FRA values were not statistically significantly different but tended to be higher. The authors concluded that the underpreparation drilling technique enhances the primary implant stability, especially when the implants are inserted in low-density bone types (III and IV) [36].

García-Vives et al., 2009, in bovine ribs, in which they performed underpreparations with the osteotome technique in type IV bone, found RFA stability values in line with the results found in this work. They also showed the importance of the management of cortical bone in order to achieve greater implant stability after insertion [37].

Rodrigo et al., 2009, reported, in his study on bone ribs in bone density II and IV, higher RFA stability values in implants that were placed in sites where the cortical bone had been partially maintained. The protocol suggested for the standard preparation of the bed for the implant was the removal of the cortical burr. They found higher RFA values in all situations in which the most coronal portion of the implant site was underprepared—results in line with those found in our research [38].

In the present study, a propensity for higher ISQ values with both preparations without the cortical drill compared to the ones with it was found, although it must be taken into account that not using the cortical drill might lead to the compression of crestal bone and, consequently, bone loss around the implant. Thus, one option for improving implant stability while avoiding crestal bone loss is using the P3 technique: the underpreparation of the pilot drill while still using the cortical drill.

Despite the differences found in the methods of the clinical and in vitro studies, a strong correlation between the surgical technique and primary stability becomes evident, although there are modifying factors that should be taken in to account when choosing a surgical technique, such as the bone density. Regarding the results of this study, the following may be recommended for obtaining better primary implant stability: using the countersink procedure, avoiding the use of a cortical drill that removes the cortical layer in cases of low-density bone, and using an underpreparation technique in the most coronal aspect of the implant site.

## 5. Conclusions

Within the limitations of an in vitro study, the results showed that undersizing the implant site could be a viable method for improving implant primary stability in low-density bone sites.

Of the three types of undersizing techniques studied, the most effectives are those without a cortical drill (P2 and P4), showing that the cortical bone is good for achieving primary stability.

In view of the clinical applications of this research, it can be suggested that during the preparation of the implant site, the IT can be evaluated based on the bone density determined in a simple manner. Depending on the bone density found at the planned location for the implant, a surgical protocol should be chosen to aim for a higher stability of the implant. In the case of low bone density, the underpreparation of the implant site can be considered, especially in the most coronal portion (avoiding a cortical drill). The final stability of the implant can be verified with the RFA system. Additional in vivo studies are warranted.

## Figures and Tables

**Table 1:** Bone surface, implant surface and % of reduction for implant surface values for 3.5 and 4.0 mm diameter implants in each preparation.

**3.5 mm Implant**	**P4**	**P3**	**P2**	**P1**
Bone surface, mm^2^	90.5	95.8	101.9	103.2
Implant surface, mm^2^	103.4	90.1	51.7	41.0
Increase in implant surface compared to P1	252%	220%	126%	100%
**4.0 mm Implant**	**P4**	**P3**	**P2**	**P1**
Bone surface, mm^2^	101.9	103.2	111.2	114.6
Implant surface, mm^2^	119.4	116.9	91.6	50.6
Increase in implant surface compared to P1	252%	236%	181%	100%

**Table 2:** Values of the IT and ISQ for the 4 different types of implant preparation technique for 3.5 and 4.0 mm diameter implants. Implant Prep.—implant preparation; IT—insertion torque; RFA—resonance frequency analysis; ISQ A—ISQ first measurement; ISQ B—ISQ second measurement; ISQ X—mean of ISQ A and B; SD—standard deviation.

Implant Prep.	IT—N/cm		RFA—ISQ A		RFA—ISQ B		RFA—ISQ X	
	Mean	SD	Mean	SD	Mean	SD	Mean	SD
**VEGA 3.5 mm**								
P1	28.7	14.9	71.4	10.0	72.4	7.9	71.9	8.9
P2	27.7	16.7	77.2	6.1	77.6	6.7	77.4	6.3
P3	34.7	15.4	75.0	5.7	75.5	5.2	75.2	5.3
P4	53.8	25.5	80.8	3.7	80.8	3.4	80.8	3.4
**VEGA 4.0 mm**								
P1	37.8	20.4	73.7	7.2	73.9	6.7	73.8	6.8
P2	43.7	16.5	78.3	3.7	78.7	4.4	78.5	3.8
P3	34.6	16.3	73.9	5.0	73.6	6.3	73.8	5.4
P4	51.7	27.3	75.0	8.1	74.9	9.6	75.0	8.7

**Table 3:** Relationship between the different types of preparation (P1, P2, P3 and P4) for 3.5 and 4.0 mm diameter implants. T-Student and Mann–Whitney tests were applied if the variables showed a normal (N) or did not show normal distribution (Nn) respectively. IT—insertion torque; ISQ A—ISQ first measurement; ISQ B—ISQ second measurement; ISQ X—mean of ISQ A and B; NW—Normal preparation with cortical drill; NWO—normal preparation without cortical drill; UW—Underpreparation with cortical drill; UWO—Underpreparation without cortical drill; N—normal distribution; Nn—not normal distribution.

**3.5 mm Diameter** **Implant**	**Normal Preparation with Cortical Drill (NW) P1**				**Normal Preparation without Cortical Drill (NWO) P2**				**Underpreparation with Cortical Drill (UW) P3**				**Underpreparation without Cortical Drill (UWO) P4**			
	**IT**	**ISQ A**	**ISQ B**	**ISQ** **X**	**IT**	**ISQ A**	**ISQ B**	**ISQ** **X**	**IT**	**ISQ A**	**ISQ B**	**ISQ** **X**	**IT**	**ISQ A**	**ISQ B**	**ISQ** **X**
**Normal preparation with cortical drill (NW) P1**					NW(N) = NWO(Nn) *p* = 0.623	NW(Nn) < NWO(Nn) *p* = 0.002	NW(Nn) < NWO(Nn) *p* = 0.001	NW < NWO	NW(N) = UW(N) *p* = 0.088	NW(Nn) = UW(Nn) *p* = 0.159	NW(Nn) = UW(Nn) *p* = 0.096	NW = UW	NW(N) < UWO(N) *p* = 0.000	NW(Nn) < UWO(Nn) *p* = 0.016	NW(Nn) < UWO(Nn) *p* = 0.011	NW < UWO
**Normal preparation without cortical drill (NWO) P2**									NWO(Nn) < UW(N) *p* = 0.044	NWO(Nn) > UW(Nn) *p* = 0.020	NWO(Nn) > UW(Nn) *p* = 0.020	NWO > UW	NWO(Nn) < UWO(N) *p* = 0.000	NWO(Nn) = UWO(Nn) *p* = 0.0693	NWO(Nn) = UWO(Nn) *p* = 0.396	NWO = UWO
**Underpreparation with cortical drill (UW) P3**													UW(N) < UWO(N) *p* = 0.000	UW(Nn) = UWO(Nn) *p* = 0.117	UW(Nn) = UWO(Nn) *p* = 0.138	UW = UWO
**Underpreparation without cortical drill (UWO) P4**																
**4.0 mm diameter** **implant**	**Normal Preparation with Cortical Drill (NW) P1**				**Normal Preparation without Cortical Drill (NWO) P2**				**Underpreparation with Cortical Drill (UW) P3**				**Underpreparation without Cortical Drill (UWO) P4**			
	**IT**	**ISQ A**	**ISQ B**	**ISQ X**	**IT**	**ISQ A**	**ISQ B**	**ISQ X**	**IT**	**ISQ A**	**ISQ B**	**ISQ X**	**IT**	**ISQ A**	**ISQ B**	**ISQ X**
**Normal preparation with cortical drill (NW) P1**					NW(Nn) < NWO(N) *p* = 0.020	NW(Nn) < NWO(N) *p* = 0.001	NW(Nn) < NWO(Nn) *p* = 0.000	NW < NWO	NW(Nn) = UW(N) *p* = 0.603	NW(Nn) = UW(N) *p* = 0.689	NW(Nn) = UW(Nn) *p* = 0.965	NW = UW	NW(Nn) < UWO(N) *p* = 0.017	NW(Nn) = UWO(Nn) *p* = 0.174	NW(Nn) = UWO(Nn) *p* = 0.097	NW = UWO
**Normal preparation without cortical drill (NWO) P2**									NWO(N) > UW(N) *p* = 0.032	NWO(N) > UW(N) *p* = 0.000	NWO(Nn) > UW(Nn) *p* = 0.000	NWO > UW	NWO(N) = UW(N) *p* = 0.108	NWO(N) > UWO(Nn) *p* = 0.000	NWO(Nn)> UWO(Nn) *p* = 0.047	NWO > UWO
**Underpreparation with cortical drill (UW) P3**													UW(N) < UWO(N) *p* = 0.002	UW(N) = UWO(Nn) *p* = 0.077	UW(Nn) = UWO(Nn) *p* = 0.114	UW = UWO
**Underpreparation without cortical drill (UWO) P4**																
